# Female Genital Mutilation in COVID-19 Scenery

**DOI:** 10.34172/aim.2022.102

**Published:** 2022-09-01

**Authors:** Vitorino Modesto dos Santos, Fabiana Ruas Domingues Modesto

**Affiliations:** ^1^Internal Medicine, Armed Forces Hospital, Catholic University of Brasília-DF, Brazil; ^2^Brazilian Federation of Gynecology and Obstetrics (Febrasgo), São Paulo, SP, Brazil

## Dear Editor,

 February 6 is the International Day of Zero Tolerance against female genital mutilation (FGM), which has affected over 200 million girls and women worldwide, representing a gender-based violence that leads to physical and psychological harms.^1-6^ The burdensome social and general health adverse effects of FGM are more impressive in Africa, Middle East and Asia, but may also reach their daughters in other countries.^1-6^ Females of low-income populations and lower educational resources are more prone to suffering from the adverse consequences of FGM, because of cultural or religious involvements.^1-6^ The current pandemic has impaired the objectives of the World Health Organization Sustainable Development Goal 5.3, that focuses on reduction and elimination of FGM.^2-4,6^

 After December 2019, the attempts to control the COVID-19 pandemic with lockdowns have impaired some routine aspects of medical care such as sexual and reproductive care, favoring increased unwanted pregnancies and FGM. In general, the lockdown favors the higher number of violence against females, and one estimates that this pandemic will instigate the occurrences of two million cases of FGM.^1-4^ Acute and chronic physical and psychological disorders due to the removal of external female genitalia are common after procedures performed for non-medical purposes. Usual long-term sequels of FGM include sensations of inferiority, fear, depression, stress, and panic; disturbances of sleep and nutrition, drug addiction; and infections.^1-6^ FGM is often performed without aseptic conditions and antibiotics are not utilized; the infectious agents related to the procedure can be viruses, bacteria, fungi, or protozoa. Lack of patient information about previous FMG may lead to diagnostic challenges.^5^

 In this scenario, the authors would like to comment on the work of Shaygani et al recently published in this journal emphasizing the major targets to eradicate FMG.^6^ Favorable results will depend on closer collaboration among public health, religious, and policy areas and are estimated to occur after at least a five-year period of activities.^6^ Suggested measures include: heavy penalties for healthcare workers who perform FGM; continued medical course for healthcare professionals who assist victims of FGM; training of surgeons for high-quality gynecological reconstructive procedures; covering the costs of care and treatment for the victims; and educational measures to change cultural or religious mistakes about FGM among populations of rural areas and slums. In addition, the authors proposed national surveys to assess better solutions for FGM.^6^

 Lastly, but not the least, some comments are added about the case study of a 27-year-old African woman with severe infection by an unidentifiedagent following an FGM procedure performed by consensus one week before her transfer to Brazil.^5^ The diversity of antimicrobials employed due to the severity of clinical manifestations could have hampered the growing of pathogens in cultures of blood, sputum and urine. Notwithstanding, infection secondary to FGM was the most probable diagnosis.^5^ The authors highlighted the increased number of females living in Western countries who previously suffered FGM, and suggested that gynecologists, nurses and midwives should be informed about consequences, including differential diagnosis of infections.^5^

 The objective is enhancing the suspicion about this challenging condition.
